# Ameliorative Potential of Donepezil with or without Prednisolone in Bleomycin-Induced Pulmonary Fibrosis in Rats: Involvement of the Anti-Inflammatory, Antioxidant, and the Antifibrotic Pathways

**DOI:** 10.3390/medicina59050980

**Published:** 2023-05-19

**Authors:** Shery Mina, Dina M. Elfeky, Ahmed M. Kabel, Sabeha E. Hedya

**Affiliations:** 1 Department of Pharmacology, Faculty of Medicine, Tanta University, Tanta 31527, Egypt; 2 National Committee of Drugs, Academy of Scientific Research and Technology (ASRT), Ministry of Higher Education, Cairo 11694, Egypt

**Keywords:** pulmonary fibrosis, bleomycin, prednisolone, donepezil, rats

## Abstract

*Background and Objectives:* Bleomycin-induced pulmonary fibrosis is one of the serious complications that may limit the use of bleomycin in cancer therapy. To date, there is no effective remedy for the amelioration of this condition. Donepezil, an anti-Alzheimer’s medication, has recently been proven to exhibit potent anti-inflammatory, antioxidant, and antifibrotic effects. To the best of our knowledge, this study represents the first study designed to investigate the prophylactic effects of donepezil, either alone or in combination with the classic anti-inflammatory drug prednisolone, in bleomycin-induced pulmonary fibrosis. *Methods:* This study was carried out on fifty rats, which were divided into five equal groups: control (Saline) group; bleomycin group; bleomycin + prednisolone group; bleomycin + donepezil group; and bleomycin + prednisolone + donepezil group. At the end of the experiments, bronchoalveolar lavage was performed to evaluate the total and differential leucocytic counts. The right lung was processed to assess the oxidative stress markers, proinflammatory cytokines, NLRP3 inflammasome, and transforming growth factor–beta1. The left lung was subjected to histopathological and immunohistochemical examination. *Results:* The administration of donepezil and/or prednisolone induced a significant amelioration of oxidative stress, inflammation, and fibrosis. In addition, these animals showed a significant amelioration of the histopathological changes of fibrosis, together with a significant decline in nuclear factor kappa B (p65) immunoexpression, compared to the group treated with bleomycin alone. However, the rats treated with the donepezil/prednisolone combination showed non-significant effects on the aforementioned parameters compared to the group treated with prednisolone alone. *Conclusions:* Donepezil may emerge as a promising drug that shows significant prophylactic effects against bleomycin-induced pulmonary fibrosis.

## 1. Introduction

Pulmonary fibrosis (PF) is a chronic inflammatory lung disease with a relatively negligible response to the available medical therapies [1]. The etiology of PF is variable. It may be a part of autoimmune diseases or genetic defects, or a result of drugs toxicity, acute lung injury, or occupational and environmental exposure. In the majority of cases, the etiology is idiopathic. The idiopathic disorders are further categorized as idiopathic interstitial pneumonias, of which the most common is IPF [2]. Unfortunately, 3–5 years is the median survival rate following the diagnosis of IPF. The only solution that may pro-long the survival rate in these patients is lung transplantation [3].

PF develops following a cascade of events. First, injury of the lung epithelial cells leads to an epithelial–to-mesenchymal transition (EMT). Then, a major fibrogenic cytokine, transforming growth factor beta-1 (TGF-β1), enhances the transformation of these mesenchymal cells (fibroblasts) into myofibroblasts, which are considered crucial cells in PF [4]. In addition, inflammation leads to the transformation of several cell types into myofibroblasts, and eventually into the deposition of extracellular matrix (ECM) proteins [2].

Despite the emergence of several potential pharmacological lines of treatment for PF, there is no detectable improvement to the survival of patients [1]. The two newly developed antifibrotic drugs (pirfenidone and nintedanib) can significantly improve the respiratory functions and postpone the progression of PF. However, the clinical usage of these drugs is limited due to their high price and the increased incidence of adverse effects. Hence, the need for further research to explore new remedies for the mitigation of PF is vital [5]. 

Bleomycin (BLM) is an important chemotherapeutic glycopeptide antibiotic that is used for the treatment of many types of malignant conditions [3]. PF is the most frequently reported adverse effect of BLM in cancer patients, even in therapeutic doses [1]. BLM enhances the production of reactive oxygen species (ROS), leading to deoxyribonucleic acid (DNA) damage, injury of epithelial cells, and excessive deposition of collagen in ECM [4]. Continued lung exposure to BLM results in more collagen synthesis and the deposition of various matrix proteins, with the net result of a significant deterioration in the pulmonary functions [6].

Corticosteroids are usually used to suppress inflammation in the fibrotic lungs. This effect may be derived from their ability to affect the inflammatory cascade on the gene expression level, with a subsequent decrease in the production of the proinflammatory cytokines and significant attenuation of the inflammatory cellular infiltration of the pulmonary tissues [7]. However, the benefit of these agents is uncertain, and the issue of long-term adverse effects is a major drawback [8].

Donepezil, a centrally acting cholinesterase inhibitor (ChEI), has been used on a wide scale in the treatment of Alzheimer’s disease (AD). Donepezil has shown an anti-inflammatory role in humans and experimental animals [9]. This was evidenced by a significant attenuation of the inflammatory reaction to endotoxemia in rats after vagal nerve stimulation and the inhibition of the production of pro-inflammatory cytokines due to the acetyl choline (ACh)-mediated stimulation of the nicotinic receptors that are expressed on the surface of the macrophages, a pathway known as “Cholinergic anti-inflammatory pathway (CAIP)” [10]. These effects, together with the proven ability of donepezil to combat the effects of oxidative stress on the different body tissues and to modulate the apoptotic pathways, may render donepezil a potential candidate for an investigation of its efficacy in cases of BLM-induced PF [11,12]. In addition, the growing interest in investigating the increased incidence of cognitive disorders and Alzheimer’s disease in cancer patients receiving chemotherapy might draw attention to the assessment of the effect of anti-Alzheimer medications, such as donepezil, on chemotherapy-induced pulmonary fibrosis [13].

Donepezil was used in an attempt to introduce new drugs for the treatment of PF. The ligand-gated α7 nicotinic acetylcholine receptor (α7nAChR) is involved in the modulation of the inflammatory response [14]. It is expressed both in the CNS and in the extra-neuronal tissues as lung fibroblasts and leukocytes, which play fundamental roles in proliferation, differentiation, migration, adhesion, cell contact, angiogenesis, and tumor progression [9]. Indeed, its expression and activation in the inflammatory cells attenuate the release of pro-inflammatory cytokines such as tumor necrosis factor-α, interleukin 1β (IL1β), NF-κB, and IL-6, an effect known as the CAIP [14]. In view of the aforementioned data, the present study was designed to investigate the potential effects of donepezil and prednisolone, either alone or in combination, in a female Wistar albino rat model of PF induced by BLM, and to clarify the possible mechanisms that might underlie these effects.

## 2. Materials and Methods

### 2.1. Drugs and Chemicals

Bleomycin 15 mg vial, obtained from Celon Laboratories Ltd., Hyderabad, Telangana, India, was reconstituted with 7.5 mL saline to reach a final concentration of 2 mg/mL. Prednisolone syrup, 15 mg/5 mL, was purchased from European Egyptian Pharmaceutical Industries, Alexandria, Egypt. Donepezil hydrochloride obtained from Sigma Aldrich Co., St. Louis, MO, USA (CAS number 120011-70-3), was dissolved in distilled water. Thiopental sodium, 500 mg vial (E.I.P.I. Co., Cairo, Egypt), was reconstituted with 10 mL saline to reach a final concentration of 50 mg/mL.

### 2.2. Animals and Groups

This experiment was carried out on 50 female Wistar albino rats aged 4–8 weeks, weighing 150–200 gm, from Tanta University animal house. They were kept under similar housing conditions (temperature 23 ± 3 °C, 12 h light/dark cycle) and had free access to food and water ad libtum. The handling of the animals followed the Helsinki declaration of animal ethics and all the experimental procedures were adopted by the institutional Research Ethics Committee of Faculty of Medicine, Tanta University, Egypt (Approval No. 34453/02/21).

**Figure 1:**
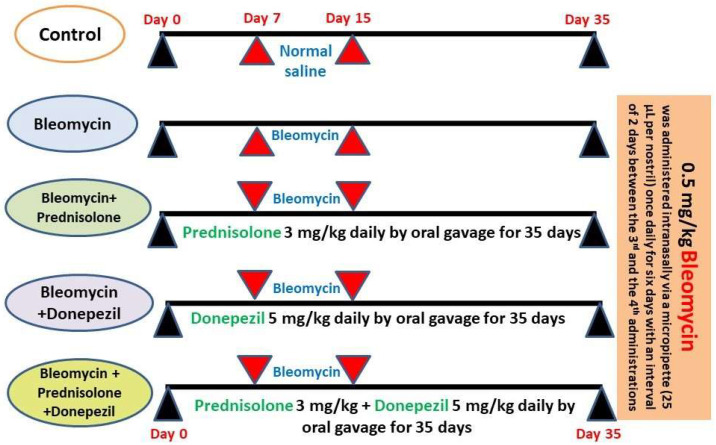
A timeline schematic diagram for the animal treatment schedule.

The animals were divided randomly into 5 equal groups of 10 rats each, as follows (Figure 1): control group in which 50 µL of normal saline was administered intranasally (IN) via a micropipette (25 µL per nostril) once daily for 6 days with an interval of 2 days between the 3rd and the 4th administrations; bleomycin group in which BLM at a dose of 0.5 mg/kg was administered IN to induce lung fibrosis via a micropipette (25 µL per nostril) once daily for 6 days with an interval of 2 days between the 3rd and the 4th administrations [15]; prednisolone group in which rats were given prednisolone 3 mg/kg daily by oral gavage [16]; donepezil group in which rats were given donepezil 5 mg/kg daily by oral gavage [17]; and prednisolone/donepezil combination group in which prednisolone was administered concomitantly with donepezil in the aforementioned doses by oral gavage. Administration of both prednisolone and donepezil started one week before, and continued for 4 weeks after, the initiation of BLM administration.

### 2.3. Lung Dissection and Bronchoalveolar Lavage (BAL) 

Twenty-one days after the end of BLM administration, the rats were euthanized with an intraperitoneal injection of thiopental (70 mg/kg) and dissected to expose the lungs. The left lung bronchus was ligated by silk sutures, then the right lung was cannulated by applying 14 G (gauge) cannula and lavaged with 6 mL ice cold saline (30 mL/kg) 4 times. Approximately 5 mL (83%) of the bronchoalveolar lavage fluid (BALF) was recovered from each rat. Then, both lungs were excised. The left lungs were used for further histopathological examination and immunohistochemical assessment. The right lungs were weighed and stored at −80 °C for evaluation of the biochemical parameters.

### 2.4. BALF Study for the Total and Differential Leucocytic Counts

BALF was centrifuged at 1000 R.P.M for 10 min. Then, the supernatants were removed. Cell pellets were re-suspended in 1 mL saline. The total and differential leucocytic counts (TLC) were determined using a hemocytometer (Marienfeld, nebular improved, Paul Marienfeld GmbH and Co. KG, Lauda-Königshofen, Germany) after staining with Turk solution.

### 2.5. Determination of Tissue Transforming Growth Factor β1 (TGF-β1), Interleukin 1 Alpha (IL-1α), Interleukin 6 (IL-6) and Nucleotide-Binding Domain-like Receptor Family, Pyrin Domain-Containing 3 (NLRP3) Inflammasome Levels

TGF-β1 was determined in the pulmonary tissues using enzyme-linked immunosorbent assay (ELISA) kits obtained from NOVA Wahan, China (Catalog No. In-Ra 1354). Kits purchased from Sigma Aldrich Co., St. Louis, MO, USA, were utilized for assessment of tissue IL-1α and IL-6 (Catalog No. RAB0272 and RAB0311, respectively). ELISA kits obtained from MyBioSource, Sand Diego, CA, USA (Catalog No. MBS2033695) were used for the quantification of NLRP3 inflammasome levels. The determination of the aforementioned parameters was achieved by following the instructions of the manufacturer.

### 2.6. Determination of the Oxidant-Antioxidant Balance in the Pulmonary Tissues 

Tissue malondialdehyde (MDA) levels were determined using lipid peroxide kits obtained from Biodiagnostic Co., Cairo, Egypt (Catalog No. MD 2529) according to the provider’s guide. Tissue superoxide dismutase (SOD), glutathione reductase (GR), and glutathione peroxidase (GPx) levels were assessed using kits obtained from Biodiagnostic Co., Cairo, Egypt (Catalog No. SD 2521, GR 2523, and GP 2524, respectively), according to the vendor’s protocol.

### 2.7. Histopathological Examination of the Pulmonary Tissues

The left lungs were immediately fixed in 10% neutral buffered formalin. After fixation, the specimens were trimmed using a scalpel to enable them to fit into an appropriately labeled tissue cassette. Processing of the tissues was performed through dehydration by immersing the lungs in increasing concentrations of alcohol to remove water and formalin. Then, xylene was used to remove the alcohol and allow infiltration with paraffin wax. After that, the lungs were infiltrated with the paraffin wax. The tissues were surrounded by a large block of molten paraffin wax, creating a “block”. Paraffin sections of 5-micron thickness were cut by the microtome and stained with hematoxylin and eosin (H&E) stain, as well as Mallory trichrome stain, and examined using a light microscope for assessment of the histopathological changes and determination of the degree of fibrosis. 

The histopathological sections were graded according to the method of Ashcroft. Each slide was graded from 0–8 (5), as follows: (0) refers to normal lung; (1) denotes minimal fibrous thickening of the alveolar or bronchiolar walls; (2–3) denotes moderate thickening of the alveolar or bronchiolar walls without obvious damage to the lung architecture; (4–5) denotes increased fibrosis with definite damage to lung structure and formation of fibrous bands or small fibrous masses; (6–7) denotes severe distortion of the lung structure and large fibrous areas; and (8) denotes total fibrous obliteration of the field [18].

### 2.8. Immunohistochemical (IHC) Detection of Nuclear Factor Kappa B (NF-κB) p65 in the Pulmonary Tissues

Sections from the lungs were taken on charged slides (4 μm thickness) and dried overnight at 60 °C. Sections were deparaffinized, rehydrated, heat-induced epitope retrieval (HIER) was performed, and tissues were boiled using Master Diagnostics EDTA buffer pH8 for 20 min at 95 °C. Upon completion, it was rinsed with 3–5 changes of distilled or deionized water, followed by cooling at room temperature for 20 min. Then, an endogenous peroxidase block was used for blocking for 10 min at room temperature using peroxidase solution. These sections were immunostained with primary antibody polyclonal IgG to rat NF-κB (p65) (Thermo Scientific Lab Vision, Catalog No. RB9034-R7), counterstained with hematoxylin, and the slides were visualized under light microscope. Calculation of the percentage of positive nuclear staining for NF-κB (p65) was performed via the IHC profiler tool in the image J software (1.49v), national institute of health. Evaluation of the NF-kB (p65) immunostaining was performed semi-quantitatively according to both the intensity and quantification of the positively stained nuclei. They were quantified as 0 (negative) when there was no nuclear immunostaining; +1 (weak positive) means positive nuclear immunostaining in 1–20% of the cells under examination; +2 (moderately positive) means positive nuclear immunostaining in 20–30% of the cells under examination; and +3 (strongly positive) refers to positive nuclear immunostaining in over 30% of the cells under examination [19].

### 2.9. Statistical Analysis

The statistical analysis of the results was performed using Graph Pad Prism version 5 for Windows, 2007, Graph Pad Software, Inc., Boston, MA, United States. The obtained results were expressed as mean ± standard error of the mean (SEM). The normal distribution of data was measured using the Shapiro-Wilk normality test. Levene’s test was used to test the homogeneity of variances. One way-ANOVA (One-way analysis of variance) followed by the Tukey test were used for comparison between the different groups. The significance of the obtained data was considered at values of *p* < 0.05.

## 3. Results

### 3.1. Donepezil with or without Prednisolone Ameliorated the Effect of BLM Administration on the Total and Differential Leucocytic Counts of BALF (Cells/mL)

**Figure 2:**
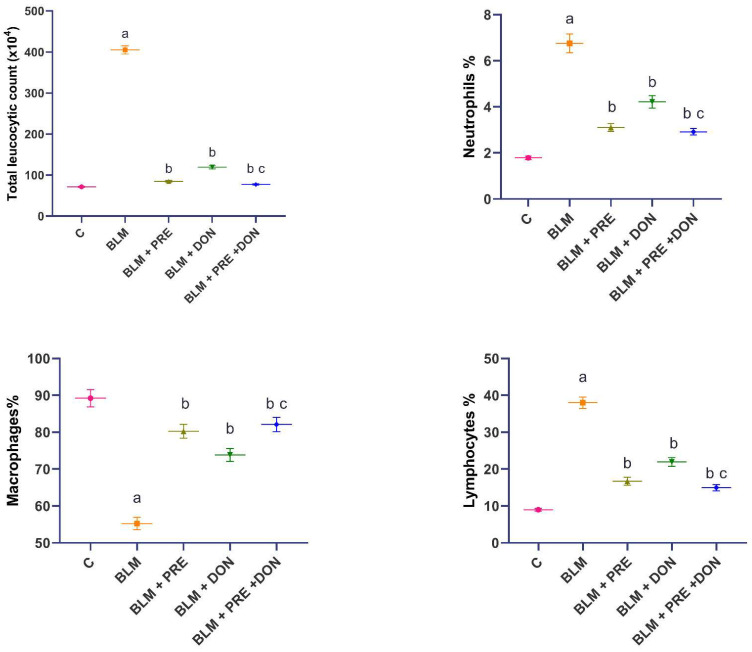
Donepezil (DON) with or without prednisolone (PRE) ameliorated the effect of BLM on the total and differential leucocytic counts in BALF (mean ± SEM). ^a^ Significant compared to the control group (*p* ˂ 0.05); ^b^ Significant compared to BLM group (*p* ˂ 0.05); ^c^ Significant compared to BLM + donepezil group (*p* ˂ 0.05).

Pulmonary fibrosis is usually associated with significant perturbations in the total and differential leucocytic counts in BALF. In the present study, BLM induced a significant increase in the total leucocytic count (TLC), neutrophils, and lymphocytes, with a significant decrease in the percentage of macrophages relative to the control group. Prednisolone and/or donepezil induced a significant decline in the TLC, neutrophils, and lymphocytes; this is associated with a significant elevation in the percentage of macrophages relative to the BLM group. The administration of the prednisolone/donepezil combination did not significantly affect the total and differential leucocytic counts when compared to the group treated with prednisolone alone (Figure 2). 

### 3.2. Donepezil with or without Prednisolone Reversed the Changes in the Tissue Levels of TGF-β1, IL-1α, IL-6, and NLRP3 Inflammasome Elicited by BLM Administration

**Figure 3:**
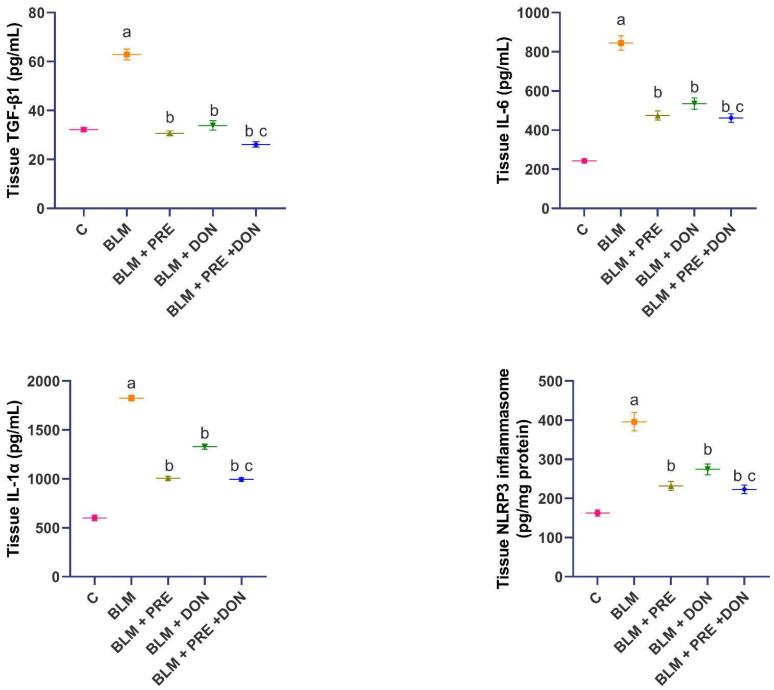
Donepezil (DON) with or without prednisolone (PRE) combatted the effect of BLM on lung tissue transforming growth factor beta 1 (TGF-β1), interleukin-1-alpha (IL-1α), interleukin-6 (IL-6), and NLRP3 inflammasome (mean ± SEM). ^a^ Significant compared to the control group (*p* ˂ 0.05); ^b^ Significant compared to BLM group (*p* ˂ 0.05); ^c^ Significant compared to BLM + donepezil group (*p* ˂ 0.05).

The changes in the TGF-β1/NLRP3 inflammasome signaling are thought to play a key role in the inflammatory events that occur in the lungs as a response to BLM administration. BLM, in the current work, induced a significant increase in tissue TGF-β1, IL-1α, IL-6, and NLRP3 inflammasome when compared to the control group. The treatment with prednisolone and donepezil, either alone or in combination, showed a significant decline in the levels of the aforementioned parameters when compared to the group treated with BLM alone. The administration of the prednisolone/donepezil combination did not significantly affect the levels of the aforementioned parameters when compared to the group treated with prednisolone alone (Figure 3). 

### 3.3. Donepezil with or without Prednisolone Restored the Pro-Oxidant/Antioxidant Balance in the Pulmonary Tissues

**Figure 4:**
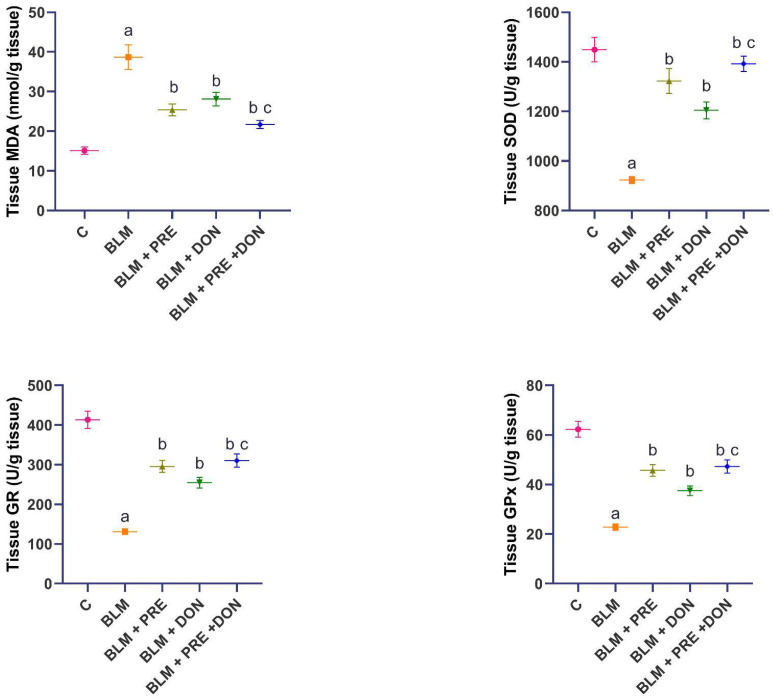
Donepezil (DON) with or without prednisolone (PRE) reversed the changes induced by BLM in lung tissue malondialdehyde (MDA), superoxide dismutase (SOD), glutathione reductase (GR), and glutathione peroxidase (GPx) (mean ± SEM). ^a^ Significant compared to the control group (*p* ˂ 0.05); ^b^ Significant compared to BLM group (*p* ˂ 0.05); ^c^ Significant compared to BLM + donepezil group (*p* ˂ 0.05).

The serious perturbations in the antioxidant defenses of the pulmonary tissues were thought to be the initiating factors for the fibrogenic events induced by BLM administration. As depicted in Figure 4, BLM in the present study induced a significant increase in the tissue MDA level and a significant decrease in the tissue SOD, GR, and GPx levels when compared to the control group. The treatment with either prednisolone or donepezil alone or in combination showed a significant decrease in the tissue MDA levels and a significant increase in the tissue SOD, GR, and GPx levels when compared to the BLM group. The levels of the aforementioned parameters were not significantly affected by the administration of the prednisolone/donepezil combination compared to the group treated with prednisolone alone.

### 3.4. Donepezil with or without Prednisolone Combatted the Effect of BLM Administration on the Histopathological Picture of the Pulmonary Tissues

**Figure 5:**
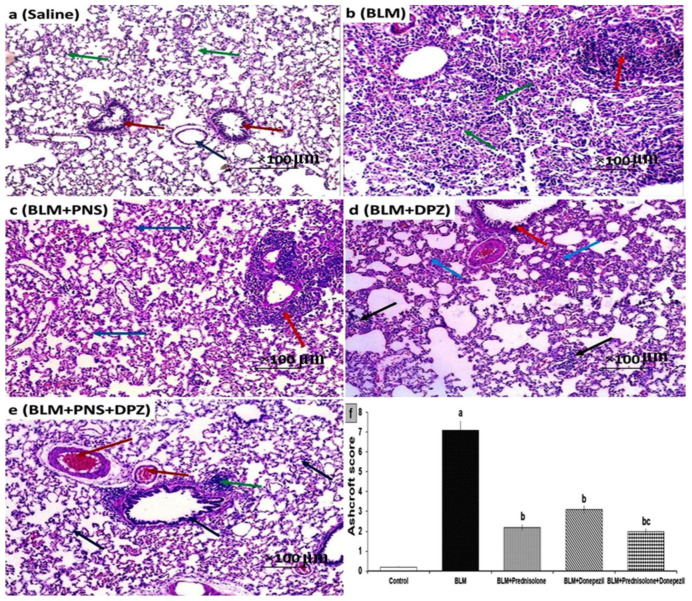
Sections of the lung (H&E × 100) from (**a**) The control (Saline) group showing normal sized alveoli with thin walls (green arrows) separated by fibrous septa containing average sized blood vessels (blue arrow) and normal sized bronchioles (red arrows); (**b**) Bleomycin (BLM) group showing alveolar severe distortion of structure and large fibrous areas (grade 7) (green arrows) surrounded by marked chronic inflammation with lymphoid follicle formation (red arrow); (**c**) Prednisolone (PNS) group showing moderate thickening of alveolar walls without obvious damage to lung (blue arrows) with focal interstitial inflammation (red arrow) (grade 2); (**d**) Donepezil(DPZ) group showing moderate thickening of alveolar walls without obvious damage to the lung (blue arrows) and moderate bronchiolar wall thickening (red arrow) (grade 3) surrounded by focal interstitial inflammation (black arrows); and (**e**) Combined group (prednisolone and donepezil) showing moderate fibrous thickening of alveolar walls and bronchiolar walls (blue arrows) (grade 2) surrounded with moderately congested vessels (red arrows) and minimal interstitial lymphocytic infiltration (green arrow); (**f**) Ashcroft score in the different studied groups (^a^ Significant compared to the control group; ^b^ Significant compared to BLM group; ^c^ Significant compared to BLM + donepezil group).

The control group showed a normal histological picture of the pulmonary tissues, with thin-walled alveoli and normal bronchioles (Figure 5a). The animals treated with BLM alone showed severe distortion of the alveolar structure, surrounded by marked chronic inflammatory cellular infiltration with lymphoid follicle formation (Figure 5b). The rats treated with either prednisolone or donepezil showed a moderate thickening of the alveolar walls, without obvious damage to the lung tissue with focal interstitial inflammation (Figure 5c,d, respectively). The group treated with the prednisolone/donepezil combination exhibited moderately congested vessels and minimal interstitial lymphocytic infiltration (Figure 5e). The changes in the Ashcroft score induced by the different treatments are depicted in Figure 5f.

**Figure 6:**
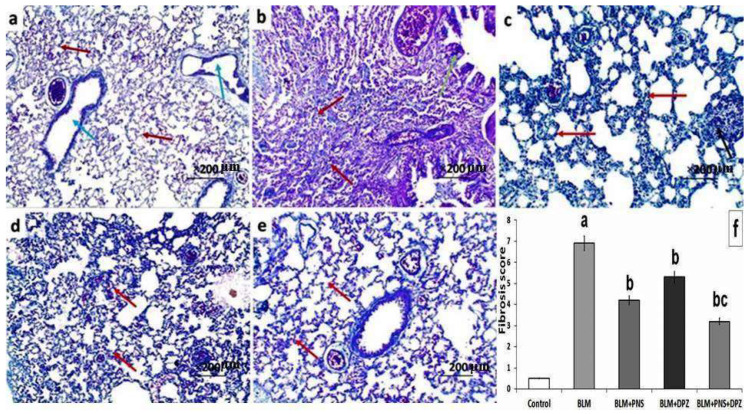
Mallory trichrome staining (×200) of lung sections from (**a**) The control (Saline) group showing normal sized alveoli with blue stained thin walls (red arrows) and blue stained thin-walled bronchioles (blue arrows); (**b**) Bleomycin group showing thick-walled blue stained alveolar fibrosis (red arrows) and blue stained thick-walled bronchioles (green arrow); (**c**) Prednisolone group showing moderate alveolar fibrosis with blue stained thick alveolar bands (red arrows) with focal interstitial inflammation (black arrow); (**d**) Donepezil group showing blue stained moderate thickening of alveolar walls (red arrows); and (**e**) Combined (prednisolone and donepezil) group showing blue stained moderate thickening of the alveolar walls (red arrows); (**f**) Fibrosis score in the studied groups (^a^ Significant compared to the control group; ^b^ Significant compared to BLM group; ^c^ Significant compared to BLM + donepezil group).

As depicted in Figure 6a, the control group showed a normal picture of the alveoli and the bronchioles, with minimal fibrous tissue in their walls. The rats treated with BLM showed large fibrous areas surrounding the alveoli and the bronchioles (Figure 6b). The rats treated with prednisolone or donepezil showed a moderate thickening of the alveolar walls, with moderate alveolar fibrosis (Figure 6c,d, respectively). The rats treated with the prednisolone/donepezil combination exhibited moderate fibrous thickening of the alveolar and the bronchiolar walls (Figure 6e). The changes in the fibrosis score induced by the different treatments are demonstrated in Figure 6f.

### 3.5. Donepezil with or without Prednisolone Mitigated the Effect of BLM Administration on the Immunohistochemical Expression of NF-κB (p65) in the Lung Tissue Sections

**Figure 7:**
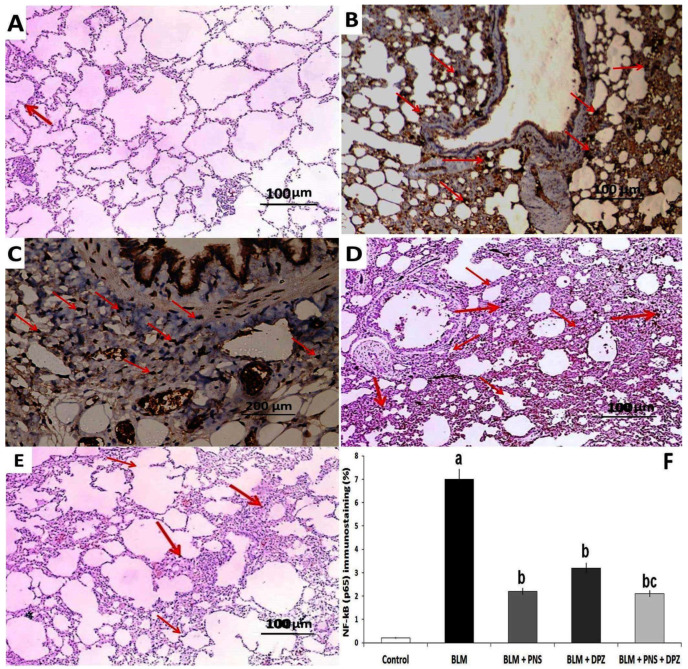
Lung sections of immunohistochemical staining NF-κB (p65) from (**A**) The control (Saline) group showing weak NF-κB (p65) staining (↑) less than 5% of the cells under examination; (**B**) Bleomycin group showing strong NF-κB (p65) staining (↑) in more than 50% of the cells under examination; (**C**) Prednisolone group showing weak NF-κB (p65) staining (↑) in 20% of the cells under examination; (**D**) Donepezil group showing moderate NF-κB (p65) staining (↑) in 25% of the cells under examination; and (**E**) Combined (prednisolone and donepezil) group showing weak NF-κB (p65) staining (↑) in less than 15% ofthe cells under examination; (**F**) The percentage of the positive NF-κB (p65) immunostaining (%) (^a^ Significant compared to the control group; ^b^ Significant compared to bleomycin group; ^c^ Significant compared to bleomycin + donepezil group).

The animals treated with BLM alone exhibited strong positive NF-κB (p65) staining in more than 50% of the cells under examination (Figure 7B,F). The rats treated with prednisolone showed weak positive NF-κB (p65) staining in 18% of the cells under examination (Figure 7C,F). Moderate positive NF-κB (p65) staining was noted in 25.1% of the cells under examination in the rats treated with donepezil (Figure 7D,F). The animals treated with the prednisolone/donepezil combination showed weak positive NF-κB (p65) staining in 17% of the cells under examination (Figure 7E,F). 

## 4. Discussion

The BLM model of lung fibrosis represents the cheapest, easiest, fastest, most reproducible, and thus the most extensively used, animal model of PF [1]. Moreover, it produces a fibrotic histologic picture similar to that of the human disease [4]. TGF-β1 is a potent profibrotic mediator that stimulates EMT. In addition, TGF-β1 promotes the transformation of fibroblasts into myofibroblasts, which create ECM and suppress its destruction [20]. Coinciding with these reports, the bleomycin group in the present study showed a significant increase in tissue TGF-β1 compared to the control group. In the present study, BLM induced a significant increase in the tissue MDA levels and a significant decline in tissue antioxidant enzymes relative to the control group. This indicated a significant increase in oxidative stress and a significant decline in the antioxidant powers, which coincided with the results of the emerging research that has proven that oxidative stress and the accumulation of ROS play a crucial role in BLM-induced pulmonary fibrosis [2,5].

The BALF in the present study, from the BLM group, showed a significant increase in the TLC, with significant perturbations of the differential leucocytic count compared to the control group, which was in agreement with the results of Kabel et al. [21]. They stated that alveolar injury induced by BLM leads to the recruitment of chronic inflammatory cells, with an enhancement of the inflammatory cascade in the pulmonary tissues.

The BLM administration in the current work elicited a severe distortion of the alveolar architecture associated with massive alveolar fibrosis, relative to the control group. These changes were in agreement with the histological changes that were observed in the study of Yu et al. [22]. They attributed the alterations in the lung tissue architecture induced by BLM administration to the profound damage of the AECs, which results in the release of the proinflammatory cytokines and growth factors that stimulate the proliferation of myofibroblasts, the secretion of a pathologic extracellular matrix, and the distortion of the alveolar architecture [4].

In the present study, BLM induced a significant increase in the percentage of positive NF-κB (p65) immunostaining in the lung tissues, which was associated with a significant elevation of the tissue levels of IL-1α, IL-6, and NLRP3 inflammasome relative to the control group. This was in accordance with the results of Cao et al. [23], who reported that BLM enhances the expression of NF-κB by the pulmonary epithelial cells, with the subsequent activation of the pathways related to NLRP3 inflammasome and with a significant increase in the production of the proinflammatory cytokines.

The administration of prednisolone to BLM-treated rats in the present study induced a significant decrease in tissue TGF-β1 compared to the rats treated with BLM alone, which was in agreement with Yao et al. [24]. However, Rasooli et al. [16] stated that prednisolone alone could not suppress PF. The explanation of this disagreement is the timing of prednisolone administration, as early treatment significantly ameliorated lung fibrosis induced by BLM. There are some studies that have shown that, for corticosteroids to be effective, treatment needs to be started early in the progression of the disease [8]. We achieved this in the present study, where we started the prednisolone treatment one week before BLM administration. At the human level, Sleijfer [25] suggested that a short course of corticosteroids initiated early after injury can prevent lung fibrosis in patients who have developed clinically evident BLM-induced pneumonitis. Recently, Bazdyrev [26] suggested that corticosteroids may be beneficial in reducing the risk and severity of post-COVID-19 PF. 

Corticosteroids may have direct effects on oxidative stress by decreasing the number and/or activity of the cells involved in ROS production, such as the granulocytes, and through modulation of the expression of the antioxidant enzymes [7]. This is in agreement with the results of the present study, where prednisolone elicited a significant decrease in the tissue MDA levels and a significant increase in tissue antioxidant enzymes compared to the BLM group.

In the present study, the prednisolone group showed a significant decrease in the TLC of BALF compared to the BLM group. This agrees with the study of Gamad et al. [27], who attributed this to the ability of corticosteroids to suppress the inflammatory cellular migration into the lungs.

Concerning the histopathological changes, the prednisolone group in the present study showed moderate alveolar fibrosis, which agrees with the study of Yu et al. [22], who revealed the role of glucocorticoids (GCs) in affecting the fibrotic pathways, inhibiting fibroblast proliferation, and in the amelioration of collagen deposition.

In the present study, prednisolone induced a significant decrease in the percentage of positive NF-κB (p65) immunostaining in the pulmonary tissues, with significant detriment to the tissue levels of IL-1α, IL-6, and NLRP3 inflammasome when compared to the BLM group. Prednisolone binding to the positive Glucocorticoid response element (GRE) forms a complex that activates the transcription of the anti-inflammatory proteins, including the inhibitors of NF-κB [8]. Through the trans-repression mechanism, binding to the negative GRE forms a complex that inhibits the transcription of inflammatory transcription factor proteins such as NF-κB and NLRP3 inflammasome. This process may be responsible for the major anti-inflammatory and immunosuppressive effects of corticosteroids [7]. 

ChEI has been shown to reduce the deposition of collagen types I and III through the inhibition of the TGF-β1/TGF-β-activated kinase1 signaling pathway via the type 2 muscarinic receptor [28]. Moreover, donepezil inhibits the TGF-β1/Smad2 signaling pathways via α7nAChR in heart transplant in rats [10]. In addition, donepezil might suppress NF-κB/NLRP3 inflammasome signaling, with a subsequent decrease in the TGF-β1 levels [14]. In this context, the donepezil administration in the current study elicited a significant decrease in TGF-β1, IL-1α, IL-6, and the NLRP3 inflammasome level compared to the BLM group.

In the current study, the donepezil group also showed a significant antioxidant effect by decreasing the tissue MDA levels and increasing the tissue antioxidant enzymes relative to the BLM group. These results agree with those of Obafemi et al. [28], who reported increased antioxidant enzyme activities in the brain tissue in response to donepezil. The accumulating data report that donepezil may protect the neurons against oxidative injury through the prevention of free-radical-mediated neuroinflammation, as donepezil possesses free radical scavenging activity [10]. 

To the best of our knowledge, the present study is one of the leading studies assessing the effect of donepezil on the total and differential leucocytic counts of BALF, where donepezil induced a significant decrease in the TLC, neutrophils, and lymphocytes, which is associated with a significant elevation in the percentage of macrophages of BALF compared to the BLM group. These findings were supported by the results of Van Westerloo et al. [29], who reported that the activation of the CAIP reduced pancreatitis severity, while vagotomy increased the local and systemic markers of inflammation and worsened acute pancreatitis, indicating that the vagus nerve exerts a tonic anti-inflammatory effect. In addition, Guo et al. [30] indicated that the inhibition of Th17 cells is a possible mechanism by which donepezil ameliorates the pathogenic changes of pulmonary fibrosis. The evidence obtained from their study might present donepezil as a promising agent for the amelioration of the inflammatory and fibrotic changes in the pulmonary tissues induced by BLM. 

In the present work, donepezil induced a significant decrease in the percentage of positive NF-κB (p65) immunostaining in the lung tissues when compared to the BLM group. These results were in accordance with Kim et al. [31], who suggested that donepezil might regulate the inhibition of microglial activation by suppressing the NF-κB pathway, possibly via the inhibition of NF-κB (p65) translocation to the nucleus. The activation of α7nAChR stimulates the Janus kinase2/signal transducers and activators of the transcription 3 signaling pathway, which inhibits the transcriptional activity of NF-κB, resulting in reduced pro-inflammatory cytokine production [32]. 

Recent studies have given attention to the role of myofibroblasts in the pathophysiology of wound healing processes [33]. Myofibroblasts were considered to be the heart of BLM-induced pulmonary fibrosis, which are responsible for the deposition of the extracellular matrix proteins that represent the platform for the fibrotic processes in the lungs [34]. Coinciding with the results of the current work, BLM administration was reported to enhance TGF-β1-induced fibroblast to myofibroblast differentiation, with the end result of an excessive deposition of the collagen fibers in the pulmonary tissues [35]. The decreased expression of TGF-β1 in the pulmonary tissues, alongside the subsequent decrease in the percentage of myofibroblasts, may provide an explanation for the mitigating effects of either donepezil or prednisolone in the present study against the pulmonary fibrotic events induced by BLM administration. 

To the best of our knowledge, this is the first study to investigate the effect of the combination of prednisolone and donepezil on BLM-induced lung fibrosis and compare it with the effect of monotherapy with either prednisolone or donepezil. When compared to the rats treated with BLM alone, the group that received the combined therapy showed a significant decrease in tissue TGF-β1, IL-1α, IL-6, NLRP3 inflammasome, MDA, and the TLC of BALF, and was associated with a significant increase in tissue antioxidant enzymes with minimal fibrosis and a significant decrease in the tissue percentage of positive NF-κB (p65) immunostaining. Meanwhile, the present work showed a non-significant effect of the prednisolone/donepezil combination compared to the prednisolone monotherapy. However, when the combined treatment was compared to the donepezil group, it showed a significant effect. Therefore, we can conclude that the effect of the combined treatment with prednisolone and donepezil in BLM-induced PF was approximately equal to the effect of the treatment with prednisolone alone. This means that the effect of donepezil was markedly attenuated when combined with prednisolone. The explanation of these findings may be that donepezil is metabolized by the liver via CYP2D6, CYP3A4, and glucuronidation. Several clinical studies have indicated that CYP2D6 is inducible by corticosteroids, based on the observation that the clearance of CYP2D6 substrates (e.g., metoprolol and dextromethorphan) increases significantly during pregnancy. During the third trimester of pregnancy, CYP2D6 activity is significantly increased (>10-fold) when the plasma concentrations of cortisol increase to ~1 μM [36]. In a comparison of prednisolone-treated patients with patients without prednisolone, the mean area under the curve of midazolam was decreased, whereas the total clearance of midazolam was increased [37]. In the studies in which interactions were proven, most reports described the induction of CYP3A4 by GCs, and therefore a decrease in the blood levels of the concomitantly administered drugs. It seems that the tacrolimus dose requirements are higher in patients concomitantly on GCs. In addition to the reduction in the blood levels, an increase in the drug-induced toxicity caused by CYP induction was also reported. Rat CYP3A4 is directly regulated by GC via GR response elements and the pregnane-x receptor, which may explain the results of the present study [38].

## 5. Conclusions

In conclusion, early treatment with prednisolone significantly ameliorated lung fibrosis induced by BLM. Donepezil is a promising drug that showed significant anti-inflammatory, antioxidant, and antifibrotic effects against BLM-induced PF. The combination of prednisolone and donepezil showed an attenuation of the donepezil effect, which may be related to the enhanced metabolism of donepezil caused by prednisolone. Further studies are needed to fully explore the mechanisms by which donepezil may mitigate BLM-induced PF and to elucidate the mechanisms underlying the potential donepezil–prednisolone interactions in this context. In addition, the results of the present study should be verified in further clinical studies to assess the possibility of its clinical application. 

## Data Availability

The data are available from the corresponding author upon reasonable request.
